# Influence of diabetes mellitus and anti-diabetic drugs on pathophysiology and prognosis for primary prostate cancer

**DOI:** 10.1007/s11255-025-04974-5

**Published:** 2026-01-02

**Authors:** Mikhail Kazachok, Aleksander Ślusarczyk, Łukasz Zapała, Tomasz Piecha, Piotr Radziszewski, Piotr Zapała

**Affiliations:** Department of General, Oncological and Functional Urology, Medical University of Warsaw, Warsaw, Poland

**Keywords:** Prostate cancer, Diabetes mellitus, Antidiabetic drugs

## Abstract

**Purpose:**

Cancer diseases and type II diabetes mellitus (DM2) are today among the major health problems, particularly in developed countries. DM2 has been historically suggested to protect against the development of prostate cancer (PCa). This study aimed to explore the mechanisms of this relation and assess its impact on treatment and prognosis for patients with PCa.

**Methods:**

The consecutive literature search was performed using PubMed, Cochrane, and Google Scholar for papers published between 2015 and 2025.

**Results:**

DM2 might reduce the risk of primary PCa development with a size effect depending on the duration of DM2. Patients with type 2 diabetes often exhibit low levels of testosterone and SHBG, which has been speculated to constitute a protective effect against PCa development. On the other hand, peripheral insulin resistance might be a protective factor, given its proliferation-promoting properties. What remains, however, the backbone element of the DM–PCa prevalence relation is metformin, which has been described to modulate PCa development through activation of AMPK kinase, reducing the c-MYC oncogene and disrupting the action of androgen receptors. Finally, DM constitutes a well-known predictor of worse surgical outcomes as well as radiotherapy toxicity.

**Conclusion:**

DM is associated with a modestly lower incidence of prostate cancer, likely mediated by hormonal cross-talk and metabolic changes. Metformin may confer additional protection in a wide range of molecular mechanisms. Nevertheless, diabetes worsens treatment course—raising surgical morbidity and radiotherapy toxicity—necessitating tight metabolic control and thoughtful anti-diabetic drug selection.

## Introduction

Cancer diseases and type II diabetes mellitus (DM2) are today among the major health problems, particularly in developed countries. The number of patients with obesity and DM2 has shown a consecutive and significant increase in recent decades, and the trend is likely to continue in the upcoming years [1]. DM2 has been speculated to increase the risk of developing different types of cancer, including liver, pancreatic, endometrial, colorectal, post-menopausal breast cancer, melanoma, and kidney cancer [1–3]. However, in the case of prostate cancer (PCa), conflicting results have been reported. The first studies on the topic have suggested that the occurrence of DM2 might protect against the development of PCa [4]. Nonetheless, today the correlation appears to be no longer straightforward. The following trends are of particular importance nowadays since PCa is the most common non-skin cancer diagnosed among men [5]. In the present narrative review, we describe the possible connections between pathological processes caused by DM2, such as altered levels of androgens, leptin, and insulin resistance, DM2-specified drugs, and its clinical effects on PCa patients, including influence on radical prostatectomy, radiotherapy, and PCa prognosis. Literature for this narrative review was selected by searching PubMed and including studies from the last 25 years with keywords ‘prostate cancer’ and ‘diabetes mellitus’.

## Diabetes mellitus in PCa patients—protective factor or trigger?

It has been believed that chronic hyperglycemia can lead directly to the proliferation of cancer cells [6]. The meta-analysis from the 2025 year (4,996,352 patients were included) suggests that in hyperglycemic patients, the risk of cancer development is increased even in the prediabetes (fasting glucose level: 100–125 mg/dL; 2 h after 75-g glucose load: 140–199 mg/dL or glycated hemoglobin level: 5.7%–6.4%) [7, 8]. In a study from 2022, with the help of syngeneic mouse tumor models of lung carcinoma and melanoma cells, it was detected that oxidative stress, caused by hyperglycemia, in endothelial cells leads to increased von Willebrand Factor expression through the transcription factor GATA1 [9]. It was also identified that high glucose levels in blood defend pancreatic cancer cells from NK (natural killers) cell-induced killing by downregulating MHC class I chain-related protein A/B (MICA/B) expression (through AMPK-Bmi1-GATA2 axis) [10].This, however, has not been proven for PCa development [11]. The relationship between the development of PCa and diabetes seems to be more complex than in several cancers for which DM2 has constituted a well-recognized risk factor. There is evidence based on numerous studies that patients with diabetes have a lower prevalence of PCa compared to age-standardized counterparts. For example, a classic meta-analysis from the 2004 year, including 14 studies published between 1971 and 2002, has shown an inverse relationship between DM2 and PCa (RR (relative risk) = 0.91, 95% CI 0.88–0.94 based on a fixed-effects model and RR = 0.91, 95% CI 0.86–0.96 based on a random-effects model) [4]. Analysis from the 2012 based on 45 studies, encompassing 8.1 million patients and 132 331 clinical cases of PCa, yielded confirmation that the baseline onset of diabetes can reduce the risk of the development of PCa (RR 0.86 for PCa prevalence in DM2 patients, 95% confidence interval (CI) 0.80–0.92) [12]. A more recent meta-analysis, summarizing 19 clinical trials, likewise found an inverse association (RR 0.84, 95% confidence interval (CI), 0.76–0.93, *P* for heterogeneity < or = 0.01) [13]. The causality and pathophysiology of this connection are, however, yet to be elucidated. We can hypothesize that among possible explanations are altered levels of androgens, leptin, and insulin resistance as well as specified medication used in diabetes mellitus treatment.

Interestingly, the relationship between PCa and DM2 might become apparent only after extending follow-up and when proper reporting is provided. It can also be limited to selected subpopulations. In a 2012 meta-analysis summarizing 25 cohort studies and 12 case–control studies (a total of 118 825 cases), the inverse relationship between the prevalence of DM2 and PCa was detected only in sensitivity analysis and was limited to population-based studies only (RR = 0.719, 95% CI (0.637, 0.812)), cohort studies from the USA (RR = 0.789, 95% CI (0.727, 0.857)) and studies with observation more than five years [14]. It might be in fact not only the issue of data consistency, but also “the protective” effect of diabetes developing with time [15]. For instance, in the study from 2013, the risk of PCa development declined with longer DM2 duration, and it was most pronounced among men diagnosed with baseline low-risk PCa (OR (odds ratio), 0.71; 95% CI (0.64–0.80)) [16]. Since the protective effect of diabetes increases with the duration of the disease, we cannot rule out that this effect may be due to effective diabetes treatment rather than diabetes itself. Consequently, a meta-analysis from the year 2013, based on 9 clinical trials, revealed that the reduction of PCa risk seems to be numerically greater in low-grade (RR 0.74, 95% CI (0.64–0.86)) and localized PCa (RR 0.72, 95% CI (0.67–0.76)), compared to high grade (RR 0.78, 95% CI (0.67–0.90)) and advanced disease (RR 0.85, 95% CI (0.75–0.97)), but without statistical significance (*P* > 0.05) [17]. It can be speculated that increasing aggressiveness can correlate with other unmodifiable factors, including germline mutations [18], race [19] and age [20] which might confound the coexisting DM2 impact or even reverse the effect, outweighing the described correlation [21]. For instance, in the meta-analysis conducted in 2012, including 1 751 274 Asian diabetic patients, an increased risk of developing PCa was observed (unadjusted RR = 2.82, 95% CI (1.73–4.58), *P* < 0.001; adjusted RR = 1.31, 95% CI (1.12–1.54), *P* = 0.001) [22]. Contradictory search results can show the heterogeneity of studied populations. Due to the fact that in different studies patients of dissimilar age, race and disease duration were examined, the results may seem controversial. Further research is needed that takes these variables into account.

## Pathophysiological interactions

### Androgens

Diabetes patients are known to present altered levels of insulin, insulin-like growth factor-1 (IGF-1), leptin, and testosterone (17). In individuals with DM2, the bioavailability of testosterone decreases compared to healthy populations, potentially impacting the development of PCa. It was observed that among diabetics, 43% had a decreased level of total testosterone, and 57% had a reduced level of free testosterone (18). Additionally, a meta-analysis from 2006 found that diabetic patients had lower mean levels of serum testosterone (difference: − 76.6 ng/dL; CI 95% − 99.4 to 53.6) (19). Interestingly, germline mutations in sex hormone-binding protein (SHBG), which lead to low levels of SHBG in the blood, were identified as independent risk factors for type 2 diabetes (20). It can be speculated that patients suffering from type 2 diabetes often exhibit low levels of testosterone and SHBG, which may provide a protective effect against the development of PCa due to its hormone-dependent nature. As a confirmation of this, we have the fact that untreated hypogonadism in men is associated with a 50% reduced risk of localized PCa and an 80% reduced risk of metastatic PCa, which was detected in a multivariate analysis from 2024 (3,222,904 patients were included) [23].

### Peripheral insulin resistance

Insulin acts as an anti-apoptotic and mitogenic factor and has been believed to promote the growth of PCa [24]. In the LNCaP (Lymph Node Carcinoma of the Prostate) xenograft model, it has been observed that mice fed with nutrients containing a significant amount of fat had increased insulin concentrations and more rapid development of PCa compared to mice on a low-fat diet. Thus, insulin resistance in prostate tissues prevents the potential carcinogenic effect of insulin. Moreover, the serum of mice containing higher amounts of fat was more mitogenic to LNCaP cells in vitro [25]. Despite this, insulin decreases the level of IGF-binding protein I, which results in a higher concentration of IGF-1 free form in the blood [26]. Thereby, insulin increases the concentration of IGF-1, which promotes PCa growth [27]. This relationship has been confirmed in experimental studies on mice [28]. Also, in a large observational study from the year 2023, it was detected that a high concentration of serum IGF-1 was associated with overall (OR per 1 SD = 1.09: 95% CI 1.07, 1.11), aggressive (1.09: 1.03, 1.16), and early-onset risk of PCa development (1.11: 1.00, 1.24) [29]. That might be crucial, due to the fact that insulin resistance might reduce the level of IGF-1 protein in blood [30]. The final clinical meaning of this phenomenon remains, however, unclear. Reduced tissue sensitivity to insulin and progressive pancreatic beta cell failure may have a paradoxical protective effect against the development of PCa. In a recent randomized 4-year controlled trial, it was observed that insulin resistance was strongly associated with reduced low-grade risk of PCa (OR, 0.84; 95% CI 0.74–0.94; *P* = 0.003), which could be explained by two possible mechanisms: (1) elimination of screening bias by performing biopsy for all patients regardless of PSA baseline levels, (2) testosterone reduces high levels of insulin, which can serve as a defense mechanism only against the initiating of low-grade PCa [31].

### Leptin resistance

DM2 has been classically associated with defective production and secretion of leptin, regardless of the metabolic balance of the disease [32]. However, in recent studies, such as in the 2024 year meta-analysis (1879 patients), it was detected that in the course of diabetes, we can observe elevated levels of leptin [33]. This allows us to assume that among diabetic patients, we will observe a state of leptin resistance, as in the case of insulin. Leptin, as a potential tumorigenic factor, might be triggered by obesity (high-calorie consumption and sedentary lifestyle) and testosterone, influencing the differentiation of PCa cells [34]. Leptin receptors are abundantly expressed in healthy and malignant tissues of the prostate [35] In vitro, leptin is a potent growth stimulator of all three PCa cell lines (LNCaP, DU145, and PC-3) [36]. Specified single nucleotide polymorphism in the leptin receptor gene (rs1137100, exon 4) was strongly associated with PCa-specific mortality [37]. On the clinical level, the association of leptin with PCa is inconsistent. In the meta-analysis of 31 observational studies, leptin had no association with overall PCa (pooled OR 1.00, 95%CI 0.98–1.02, per 2.5 ng/ml increase). Some tendency for prevalence association was observed in sensitivity analysis for cross-sectional studies only (OR 1.19, 95% CI 1.13–1.26), and there was weak evidence of a positive association with aggressive disease (OR 1.03, 95%CI 1.00–1.06) [38].

### Metformin and other antidiabetic drugs

**Fig. 1:**
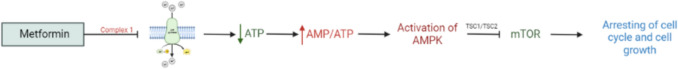
Metformin influence on cancer cells (Created in BioRender.com)

Metformin has been consistently suggested to inhibit oxygen consumption via mitochondrial complex I [39, 40]. This leads to the inhibition of oxidative respiration, which in turn reduces the amount of adenosine triphosphate (ATP) available in the cell. The resulting increase in adenosine monophosphate (AMP)/ATP ratio subsequently activates liver kinase B1 (LKB1) and 5’AMP-activated protein kinase (AMPK) [40, 41]. AMPK activation leads to a decrease in the carcinogenic activity of the mammalian target of rapamycin (mTOR) kinase, mediated by tuberous sclerosis 1 (TSC1) and tuberous sclerosis 2 (TSC2) complexes, thereby causing apoptosis and cell death [42]. (Fig. 1).

It has also been shown that activation of AMPK kinase directly inhibits endothelial cell proliferation by upregulating the expression of p21 and p27, subsequently inhibiting angiogenesis [43, 44]. Finally, it has been proven that metformin inhibits prostate intraepithelial neoplasia by reducing the overexpressed c-MYC oncogene. The following phenomenon is suspected to prevent the progression of PCa [45]. Metformin has also been reported to inhibit the growth of PCa cells through disrupting the action of androgen receptors (AR), which are activated with the help of IGF-1 [46]. Another study found that AR might also be targeted indirectly by metformin, which acts by breaking down the MID-1 protein complex [47]. In vitro studies show, however, that metformin inhibits the migration of both types of PCa cells: not only AR-positive but also AR-negative [48]. Some of the observational studies might serve as possible clinical validation of this theory. In the recent analysis of 767 men, it has been observed that individuals treated with metformin and statins were less likely to have a sum Gleason score of 8–10 (*P* = 0.033) and tended to have a lower percentage of pathological T stage of pT3 or higher (*P* = 0.064), compared to patients without any pharmacological therapy [49]. In the 2024 meta-analysis on 1,660,795 individuals, metformin use was associated with a reduced incidence of PCa (RR = 0.82, 95% CI 0.74–0.91). There was also an association between metformin and PCa recurrence (RR = 0.97, 95% CI 0.81–1.15), but statistical significance was not reached for that endpoint [50].

Metformin is not the only drug used in the treatment of diabetes, which raises the question of causal significance in the aforementioned studies and the potential for more complex pharmacological interactions. It is not clear whether the metformin effect is due to restoring the metabolic balance altered by diabetes, metformin’s selective mechanism of action, or both. The systematic review from 2022 including 47 studies (3,094,152 diabetic patients) showed a significant reduction of PCa risk development in thiazolidinediones (OR = 0.55, *P* = 0.04 in sensitivity analysis of randomized control trials (RCTs) only) and glucagon-like peptide-1 (GLP-1) receptor agonists' users (OR = 0.53, *P* = 0.006) [51, 52]. In a clinical study from the year 2011, the OS benefit of thiazolidinediones (and metformin) among patients with PCa has been recognized (Cox-regression, HR (hazard ratio) = 0.454 (95% CI 0.231–0.965, *P* = 0.040)) [53]. Inhibitors of dipeptidyl peptidase 4 (DPP-4 inhibitors) might also present a protective effect against PCa (RR = 72% (95% CI, 0.46–1.12, *P* = 0.14)) [54]. On the other hand, similar evidence for the protective effect of sodium-glucose transport protein 2 (SGLT-2) remains unclear [51]. Large registry data including single Mendelian randomization study [55] and large population-based cohort study [56] suggest protective properties, with overall risk reduction that might be as high as 23% [55] with HR 0.7 in short follow-up (5.6 years) [56].

## Influence of diabetes on PCa treatment

### Radical prostatectomy

Functional outcomes of surgical treatment with curative intent remain also strongly impacted by diabetes. In the recent study of Philippi et al., short-term continence (30–90 days post-surgery) was significantly compromised also when adjusted for local advancement and surgical approach (OR 0.26, 95%CI 0.07–0.86, *P* = 0.03) [57].In a previous meta-analysis reviewing a total of 5944 individuals, diabetes aggravated urinary continence as long as 12 months after radical prostatectomy (OR 0.54, 95%CI 0.36–0.81, (*P* = 0.003)) [58]. Diabetes constituted also an intuitive independent predictor of postoperative erectile dysfunction (severe erectile dysfunction OR 1.85, *P* < 0.001) [59]. In addition, diabetic patients experience a poorer response to treatment with phosphodiesterase type 5 inhibitors (PDE5i) following radical prostatectomy [60]. Also, in a 2019 study (312 patients were included), it was detected that pre-operative diabetes was a negative prognostic factor in gaining urinary continence in the first 18 months after robot-assisted radical prostatectomy (RARP) [61]. That fact was also confirmed in a 2023 cohort study (363 patients were included), where diabetes was an independent risk factor of urgent urinary incontinence after RARP [62].Apart from that, body mass index (BMI) > 25 kg/m^2^ (OR 2.48, 95% CI 1.28–4.81, *P* = 0.007) and DM2 (OR 2.54, 95% CI 1.16–5.55, *P* = 0.019) seem to be independent risk factors of early major complications after all types of radical prostatectomy (open, laparoscopic, and robotic), which was detected in multivariate analysis from 2014 [63]. Also, high BMI itself is associated with prolonged severe urinary incontinence (*P* = 0.001) after RARP [64].

### Radiotherapy

DM2 constitutes a strong predictor of post-radiation adverse events. Previous observational data on EBRT (external-beam radiotherapy)-treated cohorts revealed that patients with diabetes had a significantly higher incidence of late grade 2 gastrointestinal (GI) toxicity (28% vs. 17%, (*P* = 0.011)) and late grade 2 genitourinary (GU) toxicity (14% vs. 6%, (*P* = 0.001)), after radiotherapy, although acute morbidity did not differ significantly between patients with and without diabetes [65]. This observation was further confirmed by a prospective study from 2013, where DM2 patients had a higher risk both of developing grade 2 GU toxicity (RR 1.36, (*P* = 0.10)) and grade 3 GU toxicity (RR 2.74, (*P* = 0.04)) [66]. Not surprisingly, diabetes is also a well-recognized risk factor for erectile dysfunction among PCa patients treated with EBRT [67].

## Influence of diabetes on PCa prognosis

The meta-analysis including a total of 4 studies, evaluating the impact of diabetes on PCa treatment outcomes, with different types and grades of PCa from the year 2010, found a pooled risk ratio for long-term death from PCa with pre-existing diabetes of 1.57 (95% CI 1.12–2.20), respectively [68].

In a meta-analysis from the year 2014, which included 11 cohort studies, it was found that diabetes was associated with increased all-cause mortality (HR = 1.5, CI 95%: 1.25–1.79), PCa-specific mortality (HR = 1.26, 95% CI 1.20–1.33), and non-PCa mortality (HR = 1.83, CI 95%: 1.33–2.52) [69]. Similar results were obtained in another study from the year 2016 (17 cohort studies were included); diabetes also, like in the study above, was associated with a 29% increase in cancer mortality (RR = 1.29, 95% CI 1.22–1.38, I(2) = 66.68%) and a 37% increase in non-cancer mortality (RR = 1.37, 95% CI 1.29–1.45, *P* < 0.01, I(2) = 90.26%) [70].

### Prognosis after radical treatment for localized PCa

#### Radical prostatectomy

In a comparative study from 2010, it was detected that diabetic patients undergoing radical prostatectomy had an increased risk of high-grade tumor on biopsy (OR = 2.31, *P* = 0.001) and final pathological examination (OR = 2.22, *P* = 0.002) [71]. Apart from that, in the study including 4688 patients after radical prostatectomy, it was found that diabetes was associated with PCa-specific mortality (HR = 3.06, 95% CI = 1.40–6.69) and castrate-resistant PCa (HR = 2.14, 95% CI = 1.11–4.15)[72].

#### Radiotherapy

Diabetes has been consequently speculated to increase the risk of biochemical recurrence (BCR) after radiotherapy [65–67, 73]. In a large observational study (*n* = 6722 patients with PCa) from 2012, 36 patients developed BCR out of 57 (63%) DM2 patients (who were treated with radiotherapy). The DM2 was associated with a significant increase in BCR risk within a median follow-up time of 1111 days (HR = 3.79, 95% CI 1.28–11.19, *P* value = 0.01) [73].

#### Systemic treatment

The observational study by Abdel-Rahman et al. yielded no clear conclusion regarding DM2’ impact on the outcomes of docetaxel treatment in the castration-resistant PCa (CRPCa) population (no difference for OS among diabetic and non-diabetic patients (*P* = 0.908)) [74]. In DM2’ patients with metastatic CRPCa treated with docetaxel, metformin failed to show impact on cancer-specific survival (HR = 0.96, *P* = 0.9562) or overall survival (HR = 0.94, *P* = 0.9927) [75]. It can be speculated that the potential survival advantage might be outweighed by the poor prognosis of high-volume metastatic CRPCa itself as well as docetaxel toxicity. In the case of metastatic hormone-sensitive PCa, DM2’ patients present significantly shorter overall survival (uncomplicated DM2 = 25.5 months and complicated diabetes = 23.2 months) in comparison with patients without DM2 (30.9 months), which was detected in multivariate analysis (*n* = 16,721 patients) [76].

## Diabetes as a consequence of PCa treatment

The new onset of DM2 or progression of existing DM2 is a widely recognized adverse event of conventional androgen deprivation (ADT—androgen deprivation therapy). Table 1 summarizes level 1a evidence on the association between ADT + / − and DM2onset and control.

**Table 1:** Diabetes as hormonotherapy toxicity—level 1a evidence on the association between ADT and DM

Agent	Control arm	Relative risk or hazard ratio	*P* value	Study population	Main outcome	References
Gonadotropin-releasing hormone alone (GnRH)	without ADT	RR = 1.45; 95% CI 1.36–1.54	*P* < 0.001	Studies = 8 Patients: ADT users = 65 695 non-ADT users = 91 893	Increased risk of DM2	Wang et al. [77]
GnRH + oral antiandrogen		RR 1.40, 95% CI 1.01–1.93;	*P* = 0.04		Increased risk of DM2	
Antiandrogen alone		RR 1.33, 95% CI 0.75–2.36;	*P* = 0.33		No significant association with the risk of DM	
ADT	Without ADT	RR 1.43 95% CI 1.28–1.59	*P* < 0.01	Studies = 12 Patients = 336 330	Increased risk of DM2	Swaby et al. [78]
GnRH alone	Without ADT	Adjusted HR 1.44	*P* < 0.001	Patients = 73 196	Increased risk of DM2	Keating et al. [79]
Orchiectomy		Adjusted HR 1.34	*P* < 0.001			
GnRH alone	Without ADT	HR 1.53 95% CI 1.38–1.71	*P* < 0.05	Patients: ADT users = 13 078 Non-ADT users = 14 502	Increased risk of DM	Drevinskaite et al. [80]
Antiandrogen alone		HR 1.02 95% CI 0.75–1.40			No significant association with the risk of DM	

*GnRH* gonadotropin releasing hormone, *ADT* androgen deprivation therapy, *RR* relative risk, *HR* hazard ratio, *DM2* diabetes mellitus

## Discussion

In this narrative review, we summarized the complex and, at times, paradoxical relationship between type 2 diabetes mellitus (DM2) and primary prostate cancer (PCa). Several large meta-analyses have suggested a modestly reduced incidence of PCa among individuals with diabetes [4, 12–14, 17], although this inverse association seems to depend on ethnicity, duration of diabetes, tumor risk category, and the quality of follow-up [15, 16, 18–22]. Long-standing DM2 is frequently accompanied by hypogonadism, lower circulating testosterone, and altered SHBG levels, which may exert a protective effect against the development of low-grade, localized disease [23, 28]. At the same time, experimental and epidemiological data implicate hyperglycaemia, insulin resistance, IGF-1 signaling, and leptin resistance in tumor biology, suggesting that diabetes may also promote PCa initiation or progression in specific settings [6, 9, 24–27, 29–33, 36–38]. These apparently divergent findings underline the heterogeneity of diabetic populations and the need to account for metabolic and hormonal context when interpreting epidemiological associations.

Antidiabetic therapy emerges as an important modifier of both PCa risk and prognosis. Metformin remains the most extensively studied agent, with preclinical evidence of anti-tumor activity via inhibition of mitochondrial complex I, activation of AMPK, interference with mTOR signaling, and down-regulation of c-MYC and androgen receptor pathways [39–48]. Observational data suggest reduced PCa incidence and, in some cohorts, more favorable pathological features or improved outcomes among metformin users, particularly in combination with statins [42, 49, 50, 53]. Beyond metformin, thiazolidinediones and GLP-1 receptor agonists have been associated with a reduced risk of incident PCa, while incretin-based drugs as a class appear to decrease PCa incidence in patients with DM2 [15, 51, 52, 54]. For DPP-4 inhibitors, most data point to a neutral or only weakly protective effect [51], and current evidence for SGLT-2 inhibitors is limited to emerging registry-based and Mendelian randomization analyses that suggest a possible risk reduction but require longer follow-up and mechanistic validation [15, 51].

From a clinical perspective, diabetes has relevant implications for treatment tolerability and outcomes in men with PCa. DM2 is associated with worse functional recovery after radical prostatectomy, including lower rates of early and long-term urinary continence and erectile function, as well as a higher risk of perioperative complications, partly mediated by excess body weight [57–64, 71, 72]. Similarly, diabetes constitutes a risk factor for late gastrointestinal and genitourinary toxicity and erectile dysfunction after external-beam radiotherapy [65–67, 73]. Meta-analyses consistently show increased all-cause and PCa-specific mortality among patients with pre-existing diabetes [68–70], whereas in metastatic disease the impact of DM2 on overall survival may be partly overshadowed by tumor burden and systemic treatment toxicity [74–76]. Finally, androgen deprivation therapy substantially increases the risk of new-onset or worsening diabetes, thereby completing a bidirectional relationship between metabolic disease and PCa [77–80]. These findings support an integrated approach in which metabolic status, choice of antidiabetic therapy, and PCa treatment strategy are considered jointly, and justify further prospective studies to clarify whether specific antidiabetic agents can be purposefully selected to optimize both oncological and metabolic outcomes.

## Conclusions

Diabetes shows an inconsistent correlation with the prevalence of PCa. The clinical cross-talk between DM2 and PCa is driven by a variety of pathophysiological mechanisms and confounded by the use of antidiabetic drugs. Metformin and other drugs not only can reverse DM2’ metabolic consequences but also act as anti-tumorigenic agents. Further research is needed to determine the efficacy of metformin as a potential antineoplastic drug.

Simultaneously, diabetes is a well-recognized predictor of post-radiation adverse events and worse functional outcomes after prostatectomy. In a metastatic disease scenario, diabetes might have a limited impact on oncological prognosis, but remains the crucial factor mitigating the treatment safety. Diabetes is also a potential adverse event of androgen deprivation therapy and should be taken into account, due to the higher risk of complications mentioned above.

## Data Availability

No datasets were generated or analyzed during the current study.
